# Nuclear Functions of TOR: Impact on Transcription and the Epigenome

**DOI:** 10.3390/genes11060641

**Published:** 2020-06-10

**Authors:** R. Nicholas Laribee, Ronit Weisman

**Affiliations:** 1 Department of Pathology and Laboratory Medicine, College of Medicine and Center for Cancer Research, University of Tennessee Health Science Center, 19 South Manassas, Cancer Research Building Rm 318, Memphis, TN 38163, USA; 2 Department of Natural and Life Sciences, The Open University of Israel, University Road 1, Ra’anana 4353701, Israel

**Keywords:** target of rapamycin, TORC1, TORC2, epigenetics, histones, acetylation, methylation, transcription, genomic stability, cancer

## Abstract

The target of rapamycin (TOR) protein kinase is at the core of growth factor- and nutrient-dependent signaling pathways that are well-known for their regulation of metabolism, growth, and proliferation. However, TOR is also involved in the regulation of gene expression, genomic and epigenomic stability. TOR affects nuclear functions indirectly through its activity in the cytoplasm, but also directly through active nuclear TOR pools. The mechanisms by which TOR regulates its nuclear functions are less well-understood compared with its cytoplasmic activities. TOR is an important pharmacological target for several diseases, including cancer, metabolic and neurological disorders. Thus, studies of the nuclear functions of TOR are important for our understanding of basic biological processes, as well as for clinical implications.

## 1. Introduction

Signal transduction pathways act to communicate extracellular and intracellular signals to effector proteins that control a range of fundamental cellular processes. The discovery of the TOR (target of rapamycin) serine/threonine protein kinases, first in yeast cells and later in human cells, revealed TOR-dependent signaling pathways that control metabolism, proliferation, and survival in response to nutrients, energy status, growth factors, and hormones (reviewed in [1,2,3,4]). While TOR studies initially focused on the cytoplasmic functions of TOR, accumulating data suggests that TOR is also active in the nucleus, where it directly affects DNA- and chromatin-dependent functions. Several reviews have already highlighted the nuclear functions of TOR [5,6,7]. Here, we will survey recent studies, while focusing on both the mammalian TOR (mTOR), as well as the yeast TOR proteins in the two evolutionarily distant yeast model systems—the budding yeast, *Saccharomyces cerevisiae* (*Sc*TOR1 and *Sc*TOR2) and the fission yeast, *Schizosaccharomyces pombe* (*Sp*Tor1 and *Sp*Tor2). 

TOR is an atypical serine/threonine protein kinase that belongs to the family of phosphatidylinositol-kinase-related kinases (PIKKs). Other members of the family include ATM (ataxia-telangiectasia mutated) and ATR (ATM and Rad3-related) which, like TOR, are also conserved from yeast to human. ATM and ATR have traditionally been considered nuclear proteins that function in response to genotoxic stress, but more recent studies established that they also have cytoplasmic functions [8,9]. Interestingly, TOR studies have undergone an opposite direction of research focus. TOR proteins were first thought to be cytoplasmic, with a critical role in regulating protein synthesis [10,11]. However, as detailed below, TOR and its closely associated protein interactors and direct downstream effectors also localize to the nucleus where they regulate critical nuclear activities involved in gene expression. 

TOR kinases form the catalytic subunit of two distinct multi-subunit complexes: TOR complex 1 and TOR complex 2 (TORC1 and TORC2) [1,2,3,4]. In mammalian cells, a single TOR protein exists, known as mTOR, while in both *S. cerevisiae* or *S. pombe* cells two TOR genes are present. The basic composition of the TORC1 and TORC2 complexes is highly conserved throughout evolution. The presence of the Raptor protein (Kog1 in *S. cerevisiae* or Mip1 in *S. pombe*) defines TORC1, while the presence of the Rictor (Avo3 in *S. cerevisiae* or Ste20 in *S. pombe*) and Sin1 (Avo1 in *S. cerevisiae* and Sin1 in *S. pombe*) proteins defines TORC2. In *S. cerevisiae*, Tor1 or Tor2 can act as the catalytic subunit of TORC1, while only Tor2 forms TORC2 [12]. In *S. pombe*, the nomenclature is somewhat confusing -Tor1 is the main catalytic subunit of TORC2, while Tor2 is the main catalytic subunit of TORC1 [13,14] reviewed in [15]. The reason for this confusion is that the *S. pombe* TOR genes were named according to the chronological order of their discovery [16], which was before the identification of the two TOR complexes [12]. A more detailed review of the structural composition of TOR complexes can be found in [17]. 

Of the two TOR complexes, TORC1 is best known for being activated in response to nutrient availability, particularly amino acids, and for its positive role in promoting growth and anabolism while simultaneously repressing catabolic and starvation responses [1,3,4,18,19]. Only TORC1 is highly sensitive to rapamycin, while TORC2 is either insensitive, or sensitive, to rapamycin only after long exposure to the drug [12,20,21,22,23]. The use of rapamycin to probe mTORC1-dependent activities has played an important role in accelerating our understanding of mTORC1 signaling, although it should be borne in mind that TORC1 executes both rapamycin-sensitive and -insensitive functions [24,25]. TOR-specific ATP-competitive inhibitors that inhibit both TORC1 and TORC2 are also available and are being tested in clinical trials [26]. Only recently, TORC2-specific inhibitors have been identified, but their characterization is still at an early stage [27]. mTORC1 promotes protein synthesis via two well-characterized cytoplasmic targets, the AGC serine/threonine p70 S6 kinase and the eukaryotic initiation translation factor 4E-binding protein 1, 4EBP1. The role of TOR in the regulation of protein synthesis, one of the most energy-demanding cellular processes, is in accord with its function as a master regulator of cellular growth. Other anabolic processes, such as transcription and lipid and nucleotide synthesis, are also critical downstream mTORC1-regulated processes. Catabolic process inhibited by TORC1 include protein turnover and autophagy [3,28]. The cellular roles of TORC1 are highly conserved in evolution from yeast to humans [1,29].

TORC2 is also a key regulator of cell growth, proliferation, and survival, but it has distinct roles that mostly do not overlap with those of TORC1. In mammalian cells, mTORC2 is predominantly activated by growth factors downstream of insulin/PI3K signaling, although growth factor-independent activation of mTORC2, including by lipid species and glucose, has also been demonstrated [30,31,32]. Recent studies have emphasized the role that mTORC2 plays in cell metabolism via its regulation of nutrient uptake, activation of metabolic enzymes, and gene transcription [31]. mTORC2 exerts its pleiotropic effects by means of phosphorylating and activating three members of the AGC kinase family—AKT, SGK1 and PKC [31]. The role of TORC2 in the activation of downstream AGC kinases is conserved in yeast cells. In *S. cerevisae*, *Sc*TORC2 phosphorylates and activates three different AGC kinase family proteins: Ypk1, its paralog Ypk2 and Pkc1 (reviewed in [33]). Activation of Ypk1 and Ypk2 by *Sc*TORC2 in response to stresses, in particular, plasma membrane stress, is essential for lipid biosynthesis, endocytosis, and actin polarization [34,35,36,37,38,39,40,41]. In *S. pombe*, TORC2 is required for the timing of the G2/M transition, survival under stress conditions, and sexual development via phosphorylation and activation of the AGC kinase Gad8 ([42,43] reviewed in [44]). The activity of *Sp*TORC2 is acutely regulated by glucose levels and osmotic stress [45,46,47] and during quiescence [48]. Interestingly, both *Sc*TORC2 and *Sp*TORC2 have been implicated in conferring resistance to genotoxic stress and in suppression of accumulation of DNA damage foci [49,50,51]. Whereas *Sp*TORC2 acts via Gad8 to regulate genome integrity and DNA damage resistance by an as yet unknown mechanism, in *S. cerevisiae* actin-related activities controlled by *Sc*TORC2-Ypk1/2 were suggested to account for the role of *Sc*TORC2 in genome integrity [49,50,51]. The differences in TORC2 cellular functions in the two yeast systems may reflect a true divergence of function or an incomplete understanding of the activities controlled by TORC2 [52]. 

TORC1 and TORC2 are regulated by distinct mechanisms and, in turn, regulate specific downstream effectors. However, the two complexes demonstrate intricate functional links, including direct and indirect negative and positive regulation. Consequently, certain cellular activities are oppositely regulated by TORC1 and TORC2, while others are coordinated to result in the same cellular output [17,53,54]. 

## 2. Subcellular Localization of TOR and Its Activity

The question of TOR’s subcellular localization has accompanied the research carried out on TOR from its early days. TOR proteins, and later TOR complexes, have been reported to localize to the cytosol, plasma membrane (PM), lysosome, Golgi, endoplasmic reticulum (ER), peroxisome, mitochondrion, nuclear envelope, and the nucleus itself [reviewed in [55]]. Much attention has been paid to the association of TORC1 and TORC2 with cellular membranes, including the localization of mTORC1 to the lysosomal membrane [56,57], and *Sc*TORC1 or *Sp*TORC1 to the vacuolar membrane which is the organelle equivalent of the lysosome [58,59]. mTORC2 localizes to the PM, Golgi, ribosomes, mitochondria, ER, endosomes, and lysosomes (reviewed in [55] and also see [60,61,62]). The localization of TORC2 to the PM has been most extensively studied in *S. cerevisiae* [38,39,63,64,65] and was also observed in *S. pombe* [66]. 

The presence of active TOR proteins in the nucleus was first controversial. This was due to the use of different cell types, the limitations of biochemical fractionation and indirect immunofluorescence techniques, as well as the lack of direct nuclear substrates [5,55,67]. As described below, numerous studies have now demonstrated that TORC1, TORC2, and both their direct upstream and downstream regulators are present in the nucleus where they are active and interact with other nuclear proteins. 

Early studies in mammalian cells demonstrated that mTOR shuttles between the cytoplasm and the nucleus [68]. Studies carried out in normal and cancerous mammalian cell lines detected the catalytic and auxiliary subunits of TORC1 and TORC2 in the nucleus, including Raptor and Rictor [69,70,71,72,73,74,75], the upstream regulator Rheb [76], and the downstream AGC family kinases S6K1 [70], SGK1, and AKT [77,78]. Furthermore, chromatin immunoprecipitation (ChIP) analyses provided direct evidence for the presence of *Sc*TORC1 and mTOR in association with gene promoters, including promoters of Pol I- and III transcribed genes [79,80,81,82]. mTOR is also enriched at Pol II genes [82,83,84]. In *S. pombe*, subcellular biochemical fractionation analysis suggested that *Sp*TORC2 and its downstream kinase, Gad8, are predominantly nuclear, and that they are present in association with the chromatin fraction, while *Sp*TORC1 is found in equal quantities in the nucleus and the cytoplasm [85]. Indirect immunostaining analyses also supported the presence of Gad8 in the nucleus [85,86].

The question as to whether TORC1 is active in nuclei has been directly addressed in several studies. mTOR isolated from nuclei was able to phosphorylate recombinant 4E-BP1 in vitro, demonstrating active nuclear mTORC1 pools [70]. Systematic analysis of mTORC1 activity in living cells was monitored by a reporter construct that contained the 4E-BP1 protein flanked by two fluorescent proteins. mTORC1-dependent phosphorylation within the 4E-BP1 domain induced a conformational change, generating a fluorescence resonance energy transfer (FRET) signal. Targeting this activity-reporter to specific subcellular locations uncovered mTORC1 activity not only in the cytosol, lysosome, and PM but also in the nucleus [87]. These studies suggest that nuclear mTORC1 activity is induced by growth factors and amino acids, similar to the induction of its activity in the cytoplasm [87]. mTORC2 activity inside living cells was studied using a different strategy, in which the mTORC2 substrate AKT was localized to different subcellular locations by rapalog (rapamycin analog)-induced heterodimer formation of FRB (FKBP12-Rapamycin-Binding domain)-FKBP12 fusion proteins. This study showed that mTORC2 activity localized to the plasma membrane, mitochondria, and endosomal vesicles; however, it did not test for localization of mTORC2 to the nucleus [88]. A more recent study detected redistribution of Sin1 and SGK1 between the nucleus and cytoplasm in response to hormonal stimulation by angiotensin II and demonstrated mTORC2-dependent SGK1 phosphorylation at a perinuclear compartment, but not at the plasma membrane, in response to hormonal stimulation [77]. As described below, the identification of TORC1 or TORC2 substrates that are predominantly nuclear, further supports the idea of active TOR pools within the nucleus. It should also be noted that the mTORC1 subunits mLst8 and Raptor recently were characterized to be part of a novel complex that lacks mTOR but instead contains the CDK9 kinase which is a critical regulator of Pol II transcription elongation [89]. This mTORC1-like (CTORC1) complex regulates the transcription of genes involved in leukemogenesis. The ability of these mTORC1 ancillary subunits to interact with non-mTOR nuclear kinases greatly expands their role in transcriptional regulation.

## 3. Cellular Roles of TOR in Transcription and Epigenetic Regulation

### 3.1. Nuclear Transcriptional Regulation by TORC1 

#### 3.1.1. RNA Polymerase I and III Transcriptional Control 

**Figure 1:**
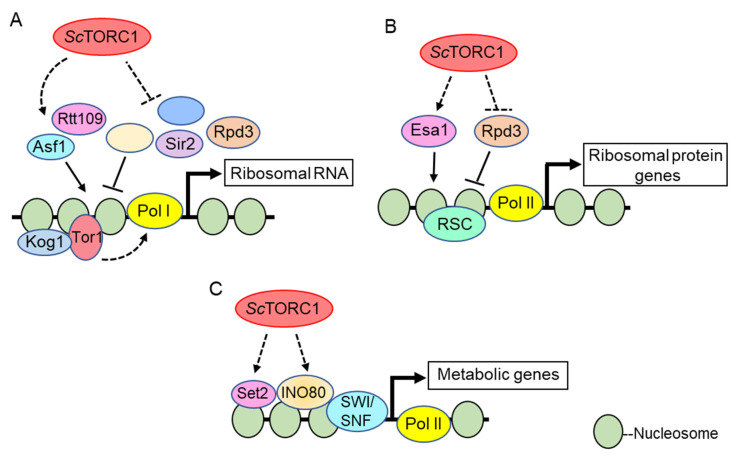
Role of the *Sc*TORC1 complex in epigenetic and transcriptional regulation. Known *Sc*TORC1 downstream epigenetic and transcriptional effectors at the rDNA (**A**), RP genes (**B**), and metabolic genes (**C**). Dashed arrows indicate regulation that is either indirect, or not yet known to be direct. Solid lines indicate direct regulation. Those pathways whose mechanisms have been described in detail are illustrated. Details are described in the text.

**Figure 2:**
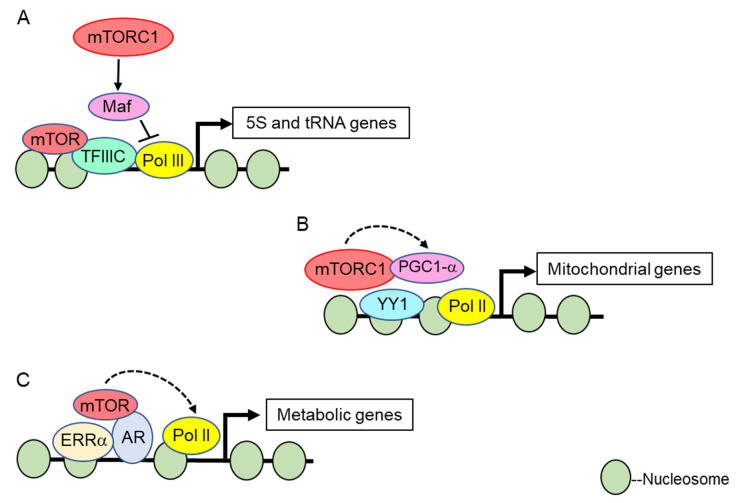
Role of the mTORC1 complex in transcriptional regulation. Known mTORC1 transcriptional roles at the 5S and tRNA genes (**A**), mitochondrial genes (**B**), and nuclear receptor activated metabolic genes (**C**). Dashed arrows indicate regulation that is either indirect, or not yet known to be direct. Solid lines indicate direct regulation. Details are outlined in the text. Only pathways whose mechanisms have been characterized in depth are represented.

To date, the best-defined mechanistic nuclear functions for TOR kinases involve their role in transcriptional regulation of the nucleolar ribosomal DNA by RNA Polymerases I and III (Pol I and Pol III) [90]. Pioneering studies in *S. cerevisiae* demonstrated that the Tor1 kinase localizes to both the cytoplasm and nucleus under nutrient replete conditions; however, upon rapamycin treatment or nutrient deprivation Tor1 becomes predominantly cytoplasmic [79]. In the nucleus, both Tor1 and Kog1 (the *Sc*TORC1 Raptor subunit) bind the promoters of the Pol I transcribed 35S rDNA and the Pol III transcribed 5S rDNA to activate transcription. Rapamycin treatment or nutrient deprivation results in Tor1 and Kog1 removal from these promoter regions, which represses both Pol I and Pol III dependent rDNA transcription [79]. Both a nuclear localization signal (NLS) and a nuclear export signal (NES) were identified in Tor1, and deletion of the NLS (but not the NES) prevented Tor1 binding to the rDNA and repressed rRNA synthesis (outlined in Figure 1A). Intriguingly, while the Tor1 NLS mutant inhibited Pol I transcription, it still allowed ribosomal protein (RP) gene transcription regulated by RNA Pol II [79]. These results were interpreted to mean that Tor1-mediated cytoplasmic activation of known RP specific transcription factors suffices to sustain RP gene transcription. How Tor1 is recruited to the rDNA is unclear. The investigators found candidate DNA binding motifs in Tor1, and mutation of these motifs disrupted Tor1 rDNA promoter binding which suggests the possibility that Tor1 binds DNA directly [79]. However, this possibility remains to be rigorously tested. Additional studies in mammalian cells demonstrated that both mTOR and Raptor interact with the DNA binding factor TFIIIC to induce Pol III-dependent transcription of 5S and tRNA genes (Figure 2A) [91]. These data further underscore the evolutionarily conserved role for nuclear TOR kinases, and possibly the *Sc*TORC1 and mTORC1 complexes, as direct Pol I and Pol III transcriptional regulators. Additional studies have detected TOR kinases and the Kog1/Raptor subunits in nucleoli which further support their direct role in Pol I and III transcription [71,92]. 

RNA Pol III transcription is also activated by TORC1 via phosphorylation of the conserved transcription repressor, Maf1, in mammalian cells [91,93,94], in *S. cerevisiae* [95,96,97] or in *S. pombe* [98]. However, the cellular localization where these phosphorylation events take place is unknown. 

#### 3.1.2. RNA Polymerase II Transcriptional Regulation

While direct TOR kinase recruitment to target genes was established at the rDNA, subsequent work in mammals demonstrated that mTOR and Raptor also contribute to nuclear Pol II transcriptional regulation. A study examining the role of muscle mTORC1 signaling showed that rapamycin treatment represses transcription of the *PGC1-α* transcriptional coactivator which activates oxidative phosphorylation and mitochondrial-specific gene expression [83]. Loss of the tuberous sclerosis complex that inhibits mTORC1 signaling enhanced *PGC1-α* transcription, thus confirming that mTORC1 activates its transcription. Both mTOR and Raptor interact with the transcription factor YY1 at mitochondrial-specific gene promoters, and rapamycin treatment prevents PGC1-α recruitment from activating transcription of these genes (Figure 2B) [83]. These data suggest that mTOR kinase activity controls the formation of a YY1/mTOR/PGC1-α multimeric transcriptional complex to enhance muscle mitochondrial activity, although the mTOR phosphorylated substrate(s) remains unknown. Recruitment of mTOR to nuclear hormone receptor-activated Pol II-transcribed genes also occurs. Genome-wide mTOR binding studies in mouse liver determined that mTOR co-regulates many of the same genes targeted by the Estrogen-related receptor alpha, ERRα, including mitochondrial TCA and lipid synthesis genes (Figure 2C) [84]. Furthermore, mTORC1 signaling stabilizes ERRα protein levels to promote this metabolic gene signature by preventing ERRα ubiquitination and proteasome-mediated degradation [84]. Whether ERRα directly binds mTOR to recruit it to these ERRα target genes, or if mTOR is recruited through an independent mechanism, remains unclear. 

mTOR also binds to the androgen receptor (AR) upon stimulation with androgens, and mTOR is recruited to a substantial number of AR-regulated genes that control cellular metabolism, including genes involved in glycolysis, oxidative phosphorylation, and lipogenesis (Figure 2C) [82]. Deregulated AR signaling drives prostate tumorigenesis, which is treated clinically by androgen ablation; however, these tumors typically evolve therapeutic resistance by AR inactivation. Intriguingly, these therapy-resistant prostate tumors exhibit an increased reliance on mTOR-dependent transcriptional activation of glycolytic and oxidative phosphorylation genes to stimulate their metabolism. Furthermore, increased nuclear mTOR in prostate tumors promotes tumorigenesis even in the absence of functional AR which correlates with worse patient survival [82]. This increased dependency on nuclear mTOR may create a metabolic vulnerability that can be therapeutically targeted to treat prostate tumors resistant to standard androgen ablation therapy. 

In yeast, less is known concerning direct Pol II transcriptional regulation by nuclear TOR kinases. The only known Pol II transcribed gene directly activated by Tor1 is *HMO1* which encodes a high mobility group box (HMGB) protein [99]. HMGBs are abundant, non-histone architectural chromatin proteins that regulate transcription. Hmo1 transcriptionally activates *Sc*TORC1-regulated genes including the RP genes [100], the 5S [100], and the 35S rDNA [100,101]. Tor1 binding to the *HMO1* promoter promotes Pol II transcription through a mechanism involving the transcription factor Ifh1 [102]. Rapamycin treatment or persistent DNA double stranded breaks inhibit *Sc*TORC1 which dissociates Ifh1 from the *HMO1* promoter to allow recruitment of the transcriptional corepressor Crf1. Subsequently, Hmo1 and Tor1 dissociate from the *HMO1* promoter to repress its transcription [99]. Intriguingly, this process requires promoter binding by the Tor1 kinase, suggesting the promoter bound Tor1 may phosphorylate a specific target to repress transcription during these stress situations.

*S. cerevisiae* genetic studies identified several additional transcriptional regulators whose disruption causes altered rapamycin sensitivity, thus implicating these factors in *Sc*TORC1 transcriptional signaling. Importantly, the RNA Polymerase II associated factor (PAF) complex that regulates both Pol II and Pol I transcription is required to properly attenuate Pol I transcription after *Sc*TORC1 inhibition [103]. The Ccr4-Not complex, which controls all stages of the mRNA lifecycle, also coordinates nutrient signaling through *Sc*TORC1 with downstream rRNA synthesis by Pol I [104]. Ccr4-Not deficient cells uncouple Pol I from *Sc*TORC1 regulation by enhancing Pol I binding to the transcription factor Rrn3 which sustains Pol I transcription initiation after *Sc*TORC1 is inhibited. Surprisingly, while *ccr4Δ* cells exhibit hypersensitivity to rapamycin, combining *ccr4Δ* with a gene deletion of a non-essential Pol I subunit mostly restores rapamycin responsiveness [104]. These data, combined with previous studies demonstrating that uncoupling Pol I regulation from *Sc*TORC1 control enhances sensitivity to *Sc*TORC1 stress [105,106], demonstrate that Pol I inhibition is essential for surviving nutrient stress conditions. 

In *S. pombe* several transcription factors that regulate the response to nitrogen starvation have been identified downstream of *Sp*TORC1 signaling including Ste11, Mei2, and Gaf1 [29]; however, there is still no indication for direct control of transcriptional regulation by nuclear *Sp*TORC1. Gaf1 is a GATA transcription factor which shuttles into the nucleus in response to nitrogen starvation in a *Sp*TORC1-dependent manner [107], similar to the regulation of the *S. cerevisiae* Gln3 and Gat1 transcription factors downstream of *Sc*TORC1 [108,109]. A global fitness profiling of *S. pombe* cells revealed a large set of deletion mutations involved in chromatin organization and gene transcription that conferred sensitivity or resistance to nutrient stress or treatment with Torin1, an ATP-competitive TOR inhibitor [110]. Further studies are required to determine the mechanisms by which these genes are regulated by TOR signaling. 

### 3.2. Epigenetic Control by TORC1 

#### 3.2.1. Histone Acetylation and Deacetylation 

In the early 2000s, *Sc*TORC1 was found to stimulate histone acetylation, which is associated with transcriptional permissiveness. In one key study, the histone acetyltransferase Esa1 was shown to bind RP gene promoters where it acetylates histone H4 to stimulate Pol II-dependent transcription through a *Sc*TORC1 dependent mechanism [111]. The Rpd3 histone deacetylase was bound to these same RP gene promoters irrespective of *Sc*TORC1 activity. After *Sc*TORC1 inhibition, Esa1 was rapidly dislodged which allowed Rpd3 to deacetylate the RP promoter chromatin and repress transcription, a process that is required for nutrient stress adaptation (Figure 1B) [111]. While *Sc*TORC1 signaling opposes Rpd3 activity at RP genes, some disagreement exists as to the exact mechanism since a different study determined that Rpd3 is not constitutively bound to these genes. Instead, Rpd3 was found to be recruited to RP promoters only after *Sc*TORC1 inhibition [112]. *Sc*TORC1 repression also results in a rapid Rpd3-mediated deacetylation of nucleolar chromatin which causes Pol I exit from nucleoli and rDNA transcriptional repression (Figure 1A) [113]. Additionally, high-throughput genetic screens identified Rpd3 to mediate transcriptional repression of *Sc*TORC1 regulated ribosome biogenesis (Ribi) gene expression during stress conditions [114]. Thus, *Sc*TORC1 signaling seems to generally oppose Rpd3-mediated histone deacetylation to promote ribosomal and Ribi gene transcription, although the exact signaling mechanisms involved have yet to be defined.

*Sc*TORC1 signaling also regulates global histone H3 lysine 56 acetylation (H3K56ac) which depends on the histone chaperone Asf1 and the acetyltransferase Rtt109 [115]. At the rDNA, Asf1-mediated H3K56ac incorporation into rDNA chromatin facilitates Pol I transcription. *Sc*TORC1 inhibition with rapamycin, or loss of the non-essential *Sc*TORC1 subunit Tco89, decreases H3K56ac [116]. Reduced rDNA H3K56ac impairs binding of the Pol I transcriptional regulators Hmo1 and the small subunit processome, leading to lower Pol I transcription and co-transcriptional ribosomal RNA processing (Figure 1A). Combining *tco89Δ* with deletion of either of the two sirtuin H3K56 deacetylases (Hst3 or Hst4) restores H3K56ac, thus providing evidence that *Sc*TORC1 represses sirtuin deacetylase activity to maintain this histone modification [116]. Subsequently, *Sc*TORC1 was shown to regulate additional histone H3/H4 acetylation modifications by repressing activation of the conserved Sit4/PP6 phosphatase complex [117]. *Sc*TORC1 inhibition increased Sit4/PP6 activity, resulting in greater Hst4 nuclear accumulation and increased Hst4 stability. This nuclear Hst4 then deacetylated H3/H4 at specific positions. While this pathway mediated some of the cellular response to *Sc*TORC1 stress, Hst4-dependent histone deacetylation had no effect on the steady-state levels of RP gene expression [117]. Therefore, the transcriptional targets actively repressed by this pathway remain unclear, although one obvious candidate would be Pol I-dependent rRNA synthesis, since these same chromatin effectors regulate Pol I transcription [116]. The sirtuin Sir2 also has a critical role in rDNA chromatin deacetylation during *Sc*TORC1 stress, which is essential for maintaining rDNA stability [118]. Collectively, these studies suggest *Sc*TORC1 signaling represses sirtuin function to maintain global histone acetylation and rDNA transcription.

Because sirtuin activity increases in the same energy-depleted environments that repress *Sc*TORC1 signaling [119], connecting *Sc*TORC1 signaling to sirtuin repression likely has co-evolved as a mechanism to limit resource consuming processes such as ribosomal transcription, and also to maintain genome stability during times of nutrient scarcity [120]. How conserved this relationship is throughout evolution remains unclear. Studies in mammals suggest mTORC1 and sirtuins are linked, although the relationship between these two pathways is less straightforward compared to yeast. For example, one study demonstrated that mTORC1 promotes glutamine anaplerosis by repressing transcription of the mitochondrial-specific sirtuin SIRT4 which inhibits glutamate dehydrogenase activity [121]. However, additional work demonstrated that mTOR enhances the activity of the sirtuin SIRT7 to stimulate Pol I and Pol III-dependent rRNA synthesis [122,123]. Therefore, while mTORC1 and sirtuins are connected in metazoans, the nature of this relationship is enigmatic and, to some extent, likely will be pathway and gene-context specific.

Histone acetylation control also contributes to mTORC1-dependent rDNA transcription in mammals. The tumor suppressor ING1 binds the rDNA and recruits the histone deacetylase HDAC1 to deacetylate histones, as well as the essential rDNA transcription factor UBF [124]. ING1-dependent HDAC1 recruitment synergizes with the nucleolar remodeling complex (NoRC) to promote rDNA heterochromatin formation and transcriptional repression. Decreased ING1 expression, which occurs in many tumors, enhances mTOR (as well as Raptor and GβL/mLst8) nucleolar localization and decreases UBF interactions with HDAC1 [124]. These changes increase UBF rDNA binding and Pol I-dependent transcription, which likely contributes to tumorigenesis by enhancing cell growth and proliferation. 

#### 3.2.2. Histone Methylation 

Site-specific histone lysine residues can be mono-, di-, and trimethylated by a variety of lysine methyltransferases to regulate gene transcription. Histone methylation does not alter the underlying lysine positive charge to affect gene expression; instead, epigenetic reader proteins containing reader modules such as chromodomains or plant homeodomain (PHD) domains selectively bind the modified lysine residue to regulate transcription [125]. The relationship between *Sc*TORC1 or mTORC1 and histone methylation is less developed compared to histone acetylation. However, recent work suggests histone methylation has an emerging role in *Sc*TORC1 and mTORC1 signaling. For example, a yeast genetic screen identified mutations in the histone H3 lysine 36 (H3K36) methylation pathway that mediates sensitivity to *Sc*TORC1 repression and nutrient stress. Loss of the sole H3K36 methyltransferase Set2, or mutation of the H3K36 residue, increased sensitivity to rapamycin in nutrient defined environments [126]. Set2-dependent H3K36 methylation binds multiple epigenetic effector complexes containing H3K36 methylation reader domains, including the RPD3S histone deacetylase [127,128], the Isw1b chromatin remodeler [129], and the NuA3b histone acetyltransferase [130]. H3K36 methylation loss causes disorganized gene body chromatin, and it increases histone exchange on transcribed genes [131]. This chromatin dysregulation leads to internal transcription from cryptic start sequences normally repressed by H3K36 methylation-dependent recruitment of RPD3S and Isw1b. Importantly, *set2Δ* reduces transcriptional fidelity and alters *Sc*TORC1-dependent nutrient stress responses by increasing bi-directional transcription initiation from gene body cryptic promoters, thus suggesting Set2 functions downstream of *Sc*TORC1 (Figure 1C) [126]. These changes also increase basal *Sc*TORC1 signaling through an unknown mechanism. One possibility for the increased *Sc*TORC1 activity could be that in cells lacking H3K36 methylation, the consequent increased cryptic transcription and disorganized chromatin generate a chromatin “signal” that somehow enhances *Sc*TORC1 signaling (discussed further in the HMGB section below). 

In mammals, H3 lysine 27 (H3K27) methylated by the Polycomb methyltransferase Ezh2 represses gene expression. In pre-adipocytes, mTORC1 signaling activates S6K1 which phosphorylates histone H2B at serine 36 (H2BS36ph) [132]. This H2BS36ph controls global Ezh2-dependent H3K27 methylation, and it also mediates Ezh2-specific H3K27 methylation at *Wnt* target genes to repress their transcription. This pathway controls the development of mature adipocytes and formation of white adipose tissue. *S6k1-/-* mice have reduced global, and *Wnt* gene specific, H3K27 methylation which prevents adipocyte development and makes these animals resistant to high-fat diet induced obesity [132]. Recently, mTORC1 also was found to phosphorylate the histone H3 lysine 9 (H3K9) demethylase JMJD1C in a nutrient-dependent manner. Phosphorylated JMJD1C then interacts with the transcription factor USF-1 to demethylate H3K9me2 at genes promoting lipogenesis in the liver, and this pathway plays a critical role in diet-induced lipogenesis [133]. Therefore, both mTORC1-dependent histone methylation and demethylation at distinct histone residues contribute to mTORC1-regulated metabolism. 

#### 3.2.3. Role of ATP-Dependent Chromatin Remodeling 

The yeast RSC (Remodeling the Structure of Chromatin) complex is an essential and abundant ATP-dependent chromatin remodeler that either activates or represses transcription through nucleosome repositioning [134]. RSC has a central role at promoters where it maintains the nucleosome free region (NFR) found at highly expressed genes controlling cell growth, including many *Sc*TORC1 regulated genes [135]. A previous study focusing on the RSC subunit Rsc9 demonstrated that *Sc*TORC1 inhibition alters Rsc9 genome-wide binding [136], thus suggesting RSC may act downstream of *Sc*TORC1 to integrate nutrient signaling with chromatin remodeling. Indeed, distinct RSC subunits were shown to either activate or repress *Sc*TORC1 regulated genes, including RP genes and the rDNA [136,137]. How these different RSC subunits accomplish this was not determined, nor is it known whether nuclear TOR kinases contribute to this regulation. However, an interesting speculation could be that nutrient signaling through *Sc*TORC1 controls the transcription initiation of a large number of growth-promoting genes via RSC-dependent NFR maintenance. In this scenario, nutrient stress would suppress *Sc*TORC1 signaling and impair RSC function. Blocking RSC activity then could allow nucleosome encroachment into the NFR, promoter occlusion, and inhibition of transcription initiation. 

Recent studies also identified the multi-subunit INO80 and SWI/SNF chromatin remodelers as effectors of *ScTORC1*-dependent transcriptional regulation [114,138,139], although the link to INO80 is mechanistically best understood. Genetic screens determined that INO80 functionally interacts with the *Sc*TORC1 pathway, and that it contributes to *Sc*TORC1-dependent metabolic gene regulation. Histone H3 lysine 18 acetylation (H3K18ac) is a histone post-translational modification dependent on active *Sc*TORC1 signaling [117], and H3K18ac co-localizes to many *Sc*TORC1-activated genes. Intriguingly, loss of the Ino80 catalytic subunit reduces H3K18ac at these genes [139], thus implicating INO80 chromatin remodeling in the regulation of a subset of *Sc*TORC1-dependent epigenetic modifications. Cells lacking functional INO80 exhibit sustained *Sc*TORC1 signaling after rapamycin treatment and are more rapamycin-resistant relative to wild-type cells [139]. How INO80 functions downstream of *Sc*TORC1, how it attenuates *Sc*TORC1 signaling, and the role this process has during nutrient stress responses, has yet to be determined (Figure 1C). 

#### 3.2.4. Role of HMGB Proteins in ScTORC1 and mTORC1 Signaling

Rapamycin-based chemical genomic screening of a histone H3/H4 mutant yeast library identified additional epigenetic pathways functionally connected to the *Sc*TORC1 pathway, including many well-characterized sites of post-translational modification [140]. These results further reinforce the concept that the full extent of *Sc*TORC1 interactions with the epigenome remains uncharacterized. Intriguingly, a histone H3 lysine 37 to alanine (H3K37A) mutation was the only mutation on either H3 or H4 that caused cell death after *Sc*TORC1 stress. This cell death consisted of both apoptosis and necrosis, and it was found to be independent of the H3K37 post-translational modification state. Instead, the H3K37A mutant reduced chromatin binding by specific HMGB proteins which resulted in their migration to the cytoplasm where they deregulate *Sc*TORC1 signaling through an unknown mechanism [141]. Reduced *Sc*TORC1 signaling is a well-established mechanism for extending chronological longevity and, consistent with this aging role, the elevated *Sc*TORC1 activity in H3K37A cells substantially reduced their longevity [141]. The increased *Sc*TORC1 signaling resembles that which occurs in *set2Δ* and INO80 mutants [126,139], thus suggesting these chromatin pathways may converge to regulate HMGB chromatin binding. In their absence, HMGBs could relocate to the cytoplasm where they deregulate nutrient signaling through *Sc*TORC1. Deregulated *Sc*TORC1 activity would make cells more sensitive to environmental stress by decreasing their ability to repress growth responses and prevent the initiation of key stress-coping mechanisms. HMGBs also contribute critical functions to *Sc*TORC1 and mTORC1-dependent transcription of RP and/or rRNA genes, including Hmo1 in yeast and UBF in mammals [100,101,142]. This HMGB dependency makes epigenetic regulation of HMGB chromatin binding a candidate mechanism by which cells connect their epigenetic status with nutrient signaling regulation.

### 3.3. Transcriptional Control by TORC2

Less is known about the roles of TORC2 in gene expression compared with TORC1. In particular, relatively little is known about the roles of *Sc*TORC2 in the regulation of gene expression. *Sc*TORC2-Ypk1 controls *ATG8* expression by repressing the stress-response transcription factors Msn2 and Msn4, thus positively regulating autophagy [143]; however, the mechanism does not appear to involve nuclear *Sc*TORC2. Of the three kinases that are targeted by *Sc*TORC2, only Pkc1, but not Ypk1 or Ypk2, has been shown to shuttle between the nucleus and the cytoplasm [144]. Pkc1 regulates the expression of B type cyclins via the phosphorylation of Ndd1, a subunit of the Mcm1-Fkh2-Ndd1 transcriptional activator, thereby controlling cell cycle progression [145]. Further studies are required to determine the role of *Sc*TORC2 in these Pkc1-dependent transcription regulation. 

**Figure 3:**
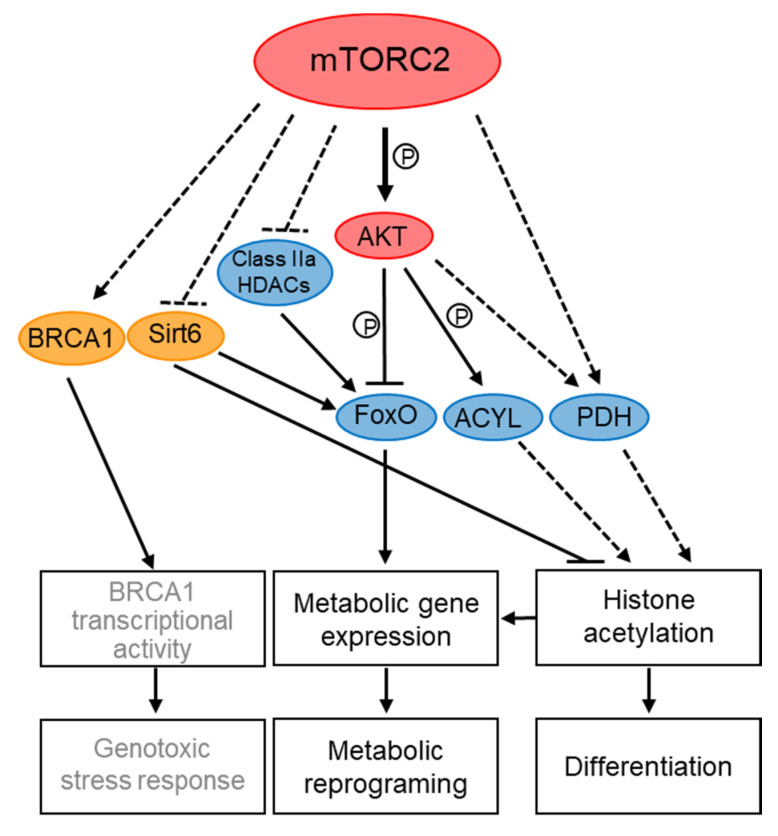
Roles of the mTORC2 complex in transcriptional and epigenetic regulation. Dashed arrows indicate regulation that is either indirect, or not yet known to be direct. Solid lines indicate direct regulation. P denotes phosphorylation. Yellow circles indicate proteins that physically interact with mTORC2. Blue circles indicate proteins that are regulated by mTORC2 or AKT outside of the nucleus. The link between mTORC2 and BRCA1 (grey letters) is more recent and yet to be tested more rigorously.

**Figure 4:**
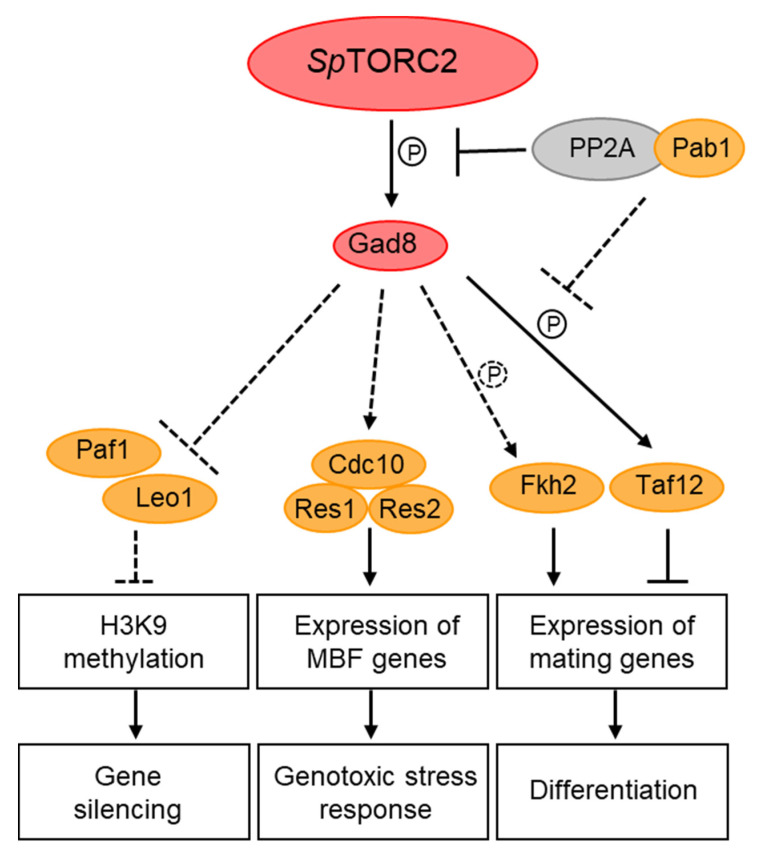
Role of the *Sp*TORC2 complex in transcriptional and epigenetic regulation. Dashed arrows indicate regulation that is either indirect, or not yet known to be direct. Solid lines indicate direct regulation. P denotes phosphorylation. The P circled with a dashed line indicates uncertainty about in vivo phosphorylation. Yellow circles indicate proteins that physically interact with Gad8. Notably, Paf1, Leo1, Taf12, and Pab1 are found primarily in the nucleus.

Better evidence for transcriptional and epigenetic control by TORC2 complexes comes from studies in mammalian and *S. pombe* cells (summarized schematically in Figure 3 and Figure 4). In mammalian cells, special attention has been given to the role of mTORC2 in transcriptional regulation of metabolism, in particular in cancer metabolic reprogramming, which drives cells into glycolysis (the Warburg effect) and increases glutaminolysis and lipogenesis [146]. mTORC2 controls metabolic processes via regulation of transcription factors, such as FoxO (Forkhead box O), SREPB1 (sterol response element-binding protein 1) and ChREBP (carbohydrate-responsive element-binding protein) (reviewed in [31,147]). These transcription factors are regulated by mTORC2 through the control of their translation rate, stability and phosphorylation which are processes that likely occur in the cytoplasm [31]. For example, mTORC2-AKT inhibits FoxO1 activity by its phosphorylation, which leads to its nuclear exclusion and reduced FoxO1-dependent transcription [147]. mTORC2 also inhibits FoxO activity in an AKT-independent manner by promoting the inactivation of class IIa histone deacetylases (HDACs), which deacetylate and thereby activate FoxO [146]. More recently, loss of mTORC2 has also been shown to cause deacetylation of FoxO1 in a sirtuin 6 (SIRT6) dependent manner, thus controlling lipid catabolism [148]. Interestingly, mTOR and Rictor co-immunoprecipitate with SIRT6, a NAD^+^-dependent deacetylase that primarily resides in the nucleus [148]. However, whether SIRT6 is regulated by mTORC2 nuclear pools is still speculative. 

mTORC2 also contributes to gene expression in response to differentiation signals during the Epithelial-to-Mesenchymal Transition (EMT), a developmental program that is enhanced by increased ribosome biogenesis and is associated with wound healing and tumor metastasis [75]. Interestingly, Rictor is localized to nuclei in response to signals that induce EMT, concomitant with an increase in rRNA synthesis, the initial step in ribosome biogenesis. During the increase in rDNA transcription, Rictor becomes associated with newly generated ribosomes, a process that in turn, leads to mTORC2 association with ribosomes [75]. Since mTORC2 is activated by ribosome association [60,149], nuclear localization of Rictor may serve as a mean to activate mTORC2 to induce the EMT developmental program, including increased Snail dependent transcription. 

In *S. pombe*, TORC2 has been implicated in the regulation of nutrient-dependent and stress-induced gene transcription, including the regulation of amino acids permeases [150], nitrogen-starvation [151] and DNA-replication stress-induced genes [85]. The MBF (Mlu-Binding Factor) complex is required for cell-cycle regulated induction of G1/S transition and DNA replication genes and it is activated by Cdk1 at the end of G1 and in response to replication stress [152]. Loss of *Sp*TORC2, or its target kinase Gad8, resulted in a decrease in the binding efficiency of the MBF complex to its cognate promoters and loss of MBF-dependent gene induction in response to DNA replication stress [85]. The inability to induce MBF transcription may explain the high sensitivity of TORC2-Gad8 mutant cells to replication stress [49]. Co-immunoprecipitation (co-IP) studies identified physical interactions between Gad8 and three of the core subunits of the MBF-transcription complex [85]. As mentioned above, Gad8 has been detected both in the cytoplasm and the nucleus [66,85,86], suggesting that Gad8 undergoes nucleocytoplasmic shuttling and further supporting nuclear functions for Gad8 in the regulation of transcription. Interestingly, mTOR has also been implicated in DNA replication stress [153]. In particular, the mTORC2 subunits Rictor, PRR5, and Sin1 have recently been shown to physically interact with the tBRCT domain of BRCA1, while the use of TOR inhibitors has suggested that mTORC2 and not mTORC1 is involved in activation of BRCA1-dependent transcription [154]. 

Other studies in *S. pombe* suggest that Gad8 is regulated via de-phosphorylation by PP2A-B55 [86], a phosphatase complex that resides mainly in the nucleus [86]. Gad8 immunoprecipitated with the regulatory subunit of PP2A, Pab1 (B55), in vivo, and PP2A-B55 de-phosphorylated in vitro the site that is targeted for phosphorylation by *Sp*TORC2 in Gad8 [86]. Under nitrogen sufficiency, *Sp*TORC1 positively regulates PP2A-B55; this in turn inhibits *Sp*TORC2-Gad8 signaling either at the level of Gad8, or downstream of Gad8 signaling [86,151,155]. The possibility that Gad8 is dephosphorylated by PP2A-B55 in the nucleus may therefore suggest a mechanistic link between cytoplasmic and nuclear regulation of TOR complexes.

Yet another physical interaction between Gad8 and a nuclear protein is its interaction with Taf12, a component of the SAGA (Spt-Ada-Gcn5 Acetyltransferase) transcriptional co-activator complex and the general transcription factor TFIID [151]. Gad8 interacts with, and phosphorylates Taf12, in response to nitrogen starvation [151]. Phosphorylation-site mutants of Taf12 suggest that phosphorylation of Taf12 by Gad8 is required for the timing of gene induction in response to nitrogen starvation and, thus, for the regulation of sexual development [151]. Furthermore, genetic analyses suggest that Gcn5, the acetyltransferase component of SAGA, acts downstream of *Sp*TORC2 [151]. Consistently, loss of Gcn5 suppressed the defects of *Sp*TORC2-Gad8 mutant cells to induce the sexual development genes *ste11*^+^ and *mei2*^+^ and partially suppressed the sterility of *gad8* mutant cells [151]. The *S. pombe* FOXO transcription factor, Fkh2 has also been implicated in regulating *ste11*^+^ induction and sexual development [156]. *Sp*TORC2-Gad8 phosphorylates Fkh2 in vitro [42], however; its phosphorylation in vivo has been controversial [42,86]. Therefore, whether Fkh2 is a target for *Sp*TORC2-Gad8 phosphorylation awaits further study. 

### 3.4. Epigenetic Control by TORC2

Metabolic signals play critical roles in determining chromatin structure [157]. Since mTORC2 is central to metabolic regulation, it is not surprising that mTORC2 also influences gene expression of metabolic genes by affecting the chromatin landscape. The effect of mTORC2 on histone modifications has been linked to changes in the availability of metabolites needed for chromatin modifications, in particular acetyl coenzyme (acetyl-CoA), which is used as the acetyl donor for all protein acetylation reactions [158,159]. mTORC2 signaling has been implicated in promoting the nuclear translocation of pyruvate dehydrogenase (PDH), leading to an increase in the level of acetyl-CoA in the nucleus and thus to changes in histone acetylation of iron metabolism genes [160] and *Snail*-dependent gene expression [161]. In addition, a recent study identified ATP-citrate lyase (ACYL), the enzyme responsible for converting citrate to acetyl CoA, as a target for mTORC2-AKT-dependent phosphorylation [162]. The mTORC2-AKT-ACYL pathway is required to stimulate histone acetylation, in particular histone H3 lysine 27 acetylation (H3K27Ac) which is a key marker of active enhancers during brown adipocytes differentiation. Consistently, promoters of key genes required for adipogenesis show low levels of H3K27Ac in Rictor knockout pre-adipocytes. Thus, mTORC2-dependent ACYL phosphorylation in the cytoplasm may lead to an overflow of acetyl-CoA that is directed towards histone acetylation in the nucleus [162]. mTORC2 additionally regulates acetyl-CoA synthesis from acetate, at least in part by controlling acetyl-CoA synthetase 2 [162], demonstrating a central role for mTORC2 in promoting acetyl-CoA synthesis.

SIRT6, which as mentioned above affects FoxO1 acetylation [148], has also been suggested to act downstream of mTORC2 in the regulation of histone H3K56Ac. Loss of mTORC2 in a model of glioma cells led to global, as well as specific reduction of H3K56Ac at the promoters of glycolytic genes, and increased recruitment of SIRT6 to these promoters [163]. Taken together, these findings call for further attention to the link between mTORC2 and SIRT6 and suggest the involvement of mTORC2 in pathways that regulate histone acetylation and de-acetylation. 

Studies in *S. pombe* implicated TORC2 in another aspect of epigenetic regulation, one that controls heterochromatin spreading and gene silencing. Genome-wide transcription analyses of *Sp*TORC2 or *gad8* disruption mutant cells indicated an extensive defect in gene expression, specifically resulting in increased non-coding RNA and subtelomeric gene transcription. Transcription profiles of *Sp*TORC2 or *gad8* mutant cells resembled the transcriptomes of cells lacking Clr3 or Clr6 which are histone deacetylase class I and II enzymes, respectively, or the transcriptome of a SWI/SNF chromatin remodeling complex RSC mutant [49,164]. These results suggest that *Sp*TORC2 is required for chromatin-mediated gene silencing. Further studies implicated *Sp*TORC2-Gad8 in chromatin-mediated gene expression and in the regulation of epigenetic states and their stability [164]. Chromatin exists in primarily two different states: as transcriptionally silent and compact heterochromatic regions, and as less condensed, transcriptionally permissive euchromatic regions. The use of a reporter gene at the mating type locus revealed the unstable nature of the epigenetic states in *Sp*TORC2-Gad8 mutant cells, as its expression stochastically alternated between repressed and expressed configurations [164]. Epigenetic instability might play an important role in the development of cancer, thus it will be of interest if this aspect of *Sp*TORC2 function is conserved in higher eukaryotes. 

Heterochromatin is characterized by histone H3 lysine-9-dimethylation (H3K9me2) [165]. The subtelomeric regions lie adjacent to the telomeric repeats and are characterized by an intermediate level of H3K9me2 that gradually declines as the heterochromatin spreads away from the telomeres [165]. The loss of gene silencing at the subtelomeric region in *Sp*TORC2-Gad8 mutant cells is accompanied by shrinkage of the H3K9me2 spreading, as demonstrated by genome-wide ChIP analysis [164]. The subtelomeric, low-level H3K9me2 region contains genes that are expressed in response to environmental changes, such as starvation [48]. Thus, *Sp*TORC2 may be involved in regulating the response to adverse conditions via chromatin-mediated gene expression of subtelomeric genes. The PAF complex is required in *S. pombe* to antagonize subtelomeric heterochromatin spreading [48,166,167]. The loss of gene silencing or subtelomeric H3K9me2 in *Sp*TORC2-Gad8 mutant cells is fully suppressed by the loss of Leo1 or Paf1, two subunits of the PAF complex [164]. Moreover, the subtelomeric region undergoes a dynamic regulation of its chromatin states in response to nitrogen starvation, which is oppositely regulated by *Sp*TORC2 and PAF [48]. Reduction of heterochromatin at subtelomeric regions following 24 hours in nitrogen starvation (early-quiescent) is correlated with reduced *Sp*TORC2 activity, while restoration of heterochromatin at this region following two weeks in nitrogen starvation (late-quiescence) correlated with re-activation of *Sp*TORC2. Thus, the finding that the loss of heterochromatic structures in early quiescence is antagonized by Leo1 inactivation suggests that PAF may act downstream of TORC2 to regulate chromatin-mediated gene expression in response to changing environmental conditions [48]. 

In conclusion, it is interesting to note that the suppression of *Sp*TORC2-associated defects by disrupting positive transcription regulators, such as PAF or Gnc5, as well as the similarities between transcription profiles of *Sp*TORC2-Gad8 mutant cells and histone deacetylases, suggest that *Sp*TORC2-Gad8 acts to restrain transcriptional permissiveness. This is in a sharp contrast with the role of *Sc*TORC1 in stimulating histone acetylation and transcriptional permissiveness (see above). Whether *Sp*TORC1 acts like *Sc*TORC1 to maintain transcriptional permissiveness via histone modifications is still unknown. Such a scenario will be particularly interesting in view of the opposite effects of *Sp*TORC1 and *Sp*TORC2 on many different cellular functions [86,151,155,168].

## 4. Unanswered Questions

### 4.1. How Are Nuclear TOR Kinases Activated?

Our current understanding of the nutrient and/or growth factor/mitogen-specific mechanisms activating TOR kinases is restricted solely to those functioning outside of the nucleus, specifically pathways that function on the cytosolic face of organelles such as vacuoles/lysosomes. How nuclear TOR kinases respond to these upstream signals remains completely unknown, so addressing this question should be a priority area for future investigation. One distinct possibility is that TOR kinases are activated in the cytoplasm, and then they shuttle to the nucleus to mediate their epigenetic and/or transcriptional effects. This possibility is supported by the observation that the TOR kinase shuttles between the cytoplasm and nucleus [68]. Another possibility could be the presence of nuclear-specific mechanisms for TOR kinase activation. A recent study determined that metazoan RagA-RagC GTPase heterodimers (which activate mTORC1 on the lysosomal surface in response to amino acid sufficiency) must transit from the nucleus to the cytoplasm to be competent for mTORC1 activation [169]. Repressing RagA-RagC nuclear entry prevents the conversion of RagC to the RagC-GDP bound state (the active signaling configuration), which inhibits mTORC1 signaling. Intriguingly, the RagC GTPase activating protein (GAP), folliculin, can localize to both the cytoplasm and nucleus [169], so one possibility could be that RagC is converted to RagC-GDP via this nuclear GAP activity. Whether the Rags and mTORC1 then interact in the nucleus to activate mTORC1 nuclear-specific functions remains an open question that will need to be addressed. 

### 4.2. Do Nuclear TOR Kinases Phosphorylate Substrates in the Nucleus?

Nuclear TOR kinases are detectable across species, yet whether these kinases phosphorylate substrates in the nucleus remains unclear. Structural studies of mTORC1 reveal that it forms dimers, and that the kinase is held in a constitutively active state [170]. Catalytic activity is regulated partially by restricting access to the ATP-binding site via the mTOR FRB domain and mLst8 subunit binding domain, while Raptor interaction further restricts access to the catalytic site to prevent phosphorylation of non-specific substrates [170]. Kinase substrate selectivity also is determined by Raptor which binds substrates through their TOR signaling (TOS) motifs [171,172,173]. TORC2 complexes have in common with TORC1 the dimeric structure that is composed of two copies of each subunit and a large central cavity [174]. Nuclear TOR kinases could form catalytically active dimeric TOR complexes to target nuclear proteins as their canonical cytoplasmic counterparts do. Alternatively, another possibility could be that nuclear TOR kinases do not form dimers. In this scenario, lack of a nuclear dimeric TOR complexes may increase substrate access to the TOR kinase catalytic site which could increase the diversity of substrates. Candidate nuclear substrates could include subunits of the Pol I, II, and III transcriptional apparatus as discussed above, or chromatin modifiers such as the p300 acetyltransferase [175]. 

Finally, nuclear TOR kinases, and the associated subunits, may provide non-catalytic functions critical for gene regulation. TOR kinases are very large, as are their Raptor and Rictor associated subunits. Their interactions with transcription factors or the transcriptional machinery on DNA may create novel surfaces which recruit additional coregulators to control gene expression. These unknowns remain critical deficiencies in our understanding TOR signaling, which are essential to address for understanding how aberrant TOR signaling causes disease.
